# Impact of Drought Stress on Photosynthetic Electron Transport in Rice Seedlings

**DOI:** 10.3390/plants15162499

**Published:** 2026-08-18

**Authors:** Zhaoqi Yu, Jin Liu, Zuxin Cheng, Renye Wu, Haiyong Weng

**Affiliations:** 1 Key Laboratory of Ministry of Education for Genetics, Breeding and Multiple Utilization of Crops, College of Agriculture, Fujian Agriculture and Forestry University, Fuzhou 350002, China; yuzhaoqi2002@163.com (Z.Y.); liujin20000606@163.com (J.L.); 2 Cross-Strait Cooperation Base on Agricultural Science and Technology Industry (Membership) Under the Ministry of Science and Technology (MOST), College of Agriculture, Fujian Agriculture and Forestry University, Fuzhou 350002, China; chengzuxin@163.com; 3 College of Mechanical and Electrical Engineering, Fujian Agriculture and Forestry University, Fuzhou 350002, China

**Keywords:** *Oryza sativa*, drought stress, photosynthesis, OJIP curve, chlorophyll fluorescence parameters

## Abstract

To investigate photosynthetic physiological response mechanisms of rice (*Oryza sativa* L.) seedling leaves under drought stress, a pot experiment with controlled watering was conducted. Four treatments were applied—control (CK), mild drought (LD), moderate drought (MD), and severe drought (SD)—to four cultivars (H17B7, HY3015, LH1H, WNSM) at seedling stage. The results showed that, after drought stress, the morphological indicators and chlorophyll content of the four rice cultivars all showed decreased trend, but a compensatory effect appeared in some varieties under the LD treatment. As drought intensified, the chlorophyll fluorescence induction kinetics (OJIP) curves’ polyphasic rise was inhibited, mainly affecting the J-I and I-P phases. Under SD, the P-phase declined by 44.36%, 36.75%, 32.61% and 50.62% compared with CK, and positive L-band, K-band, and J-band. Chlorophyll fluorescence parameters showed dynamic changes. Most chlorophyll fluorescence parameters (e.g., *F_v_*/*F_m_*) decreased, whereas *TR_o_*/*CS_o_* and *DI_o_*/*CS_o_* exhibited cultivar-dependent variations. Principal components of the chlorophyll fluorescence parameters in seedling leaves of the four rice cultivars showed differences and were clearly distinguished. Plant height, chlorophyll content, etc., are highly significantly positively correlated with *F_o_*, *DI_o_*/*CS_o_*, etc., as well as chlorophyll content with *DI_o_*/*CS_o_*. The OJIP/JIP-test responses suggest impairment of the PQ pool and downstream electron transport, with the extent of damage varying among cultivars.

## 1. Introduction

In recent years, extreme weather events have occurred more frequently, and the contradiction between water supply and demand in China has become increasingly acute [1]. According to the 2024 China Water Resources Bulletin, China’s agricultural water consumption accounts for 61.6% of the total water consumption, a decrease of 2.4 billion m^3^ compared to 2023, indicating that the water scarcity situation is becoming increasingly severe [2]. Rice (*Oryza sativa* L.) is one of the world’s major food crops, providing a staple food for more than one third of the global population [3,4]. However, this crop has an enormous irrigation water requirement, consuming more than 65% of China’s total agricultural irrigation water [5]. As a fibrous root system plant with high water requirements, rice primarily relies on adventitious roots (lateral roots) to absorb water [6,7]. This root structure causes its water absorption to depend mainly on shallow soil layers, making it more susceptible to drought [8]. Rice may encounter drought throughout its entire growth period. Existing studies have shown that drought stress causes damage to multiple physiological processes in plants, such as reducing plant growth indicators, decreasing chlorophyll content, and altering chlorophyll fluorescence parameters [9,10,11,12]. Plant photosynthesis is sensitive to drought stress [13]. In the process of photosynthetic response to environmental stress, Photosystem II (PSII) plays a crucial role [14,15]. Research indicates that under mild drought, plant leaves can protect the photosystem by consuming excess light energy through photorespiration and thermal dissipation; however, severe drought inhibits photosynthetic electron transport and reduces photosynthetic efficiency [16,17,18]. Because chlorophyll fluorescence (ChlF) can rapidly and sensitively detect changes in almost all photosynthetic processes via portable instruments, it is regarded as a “non-destructive” probe of photosynthesis and an important indicator for evaluating plant physiological status and environmental stress [19,20,21,22]. The OJIP curve refers to the fluorescence change process from the O point to the P point, which mainly reflects the primary photochemical reactions of PSII and the changes in the structure and state of the photosynthetic apparatus. The subsequent descending phase mainly reflects changes in photosynthetic carbon metabolism, with the fluorescence intensity gradually decreasing as the photosynthetic carbon metabolism rate increases. The detailed kinetics have been reported previously by Li [23]. The JIP-test, developed by Reto J. Strasser and collaborators, is a computational tool that analyzes the OJIP fluorescence transient from the perspective of various PSII reactions, allowing rapid acquisition of information on possible effects on photosynthesis, particularly on PSII and, to a limited extent, on PSI [24]. Through OJIP curve and JIP-test parameter analysis after ChlF measurement, the impact of adverse stress on the photosynthetic apparatus of plants can be determined [25,26,27].

Li et al. [28], using rapid chlorophyll fluorescence analysis, found that under drought stress during the grain-filling stage, the inhibition of PSII activity and its regulatory mechanisms varied among different rice genotypes, suggesting that these differences could serve as reference indicators for screening drought-tolerant germplasm. Xu et al. [29] elucidated the relationship between chlorophyll fluorescence parameters and canopy hyperspectral reflectance in pot-grown rice subjected to water stress at the booting stage. Through research on dry-raised rice seedlings, Lu et al. [30] demonstrated that drought stress leads to an increase in chlorophyll content and a reduction in seedling height and leaf length. Studies by Sharma et al. [31] pointed out that the chlorophyll fluorescence parameter *F_v_*/*F_m_* reflects the maximum quantum yield of PSII photochemistry, with values generally ranging from 0.75 to 0.85. Faseela et al. [32], in their stress study on rice seedling stage under PEG-simulated drought stress, pointed out that under PEG stress, *F_v_*/*F_o_* was prominently decreasing and *DI_o_*/*CS_o_* was significantly increasing. In identifying early drought in field wheat using chlorophyll fluorescence kinetics, Botyanszka et al. [33] found that the primary impact of drought was related to changes in the I-P phase of the OJIP transient.

Although chlorophyll fluorescence has been widely applied in studies on plant abiotic stress, there are still limitations. First, previous studies on rice chlorophyll fluorescence parameters have mainly focused on the tillering, booting, heading, and milk-ripe stages [34,35,36,37,38], with very limited attention paid to the seedling stage under soil-based drought conditions, which is critical for establishing stress tolerance and determining subsequent yield potential. Tolerance at the early stage of the plant influences its tolerance at the mature stage [39]. Second, most existing work has focused only on *F_v_*/*F_m_*, *DI_o_*/*RC*, *PI_abs_*, or a few other parameters rather than a more comprehensive JIP-test parameter analysis that can resolve the differential behavior of individual electron transport steps, reaction center turnovers, and energy dissipation fluxes. Therefore, we hypothesize that under different drought intensities, each cultivar exhibits distinct physiological responses, and these responses can be detected through OJIP curves and JIP-test changes. In this experiment, artificial pot-based water control was utilized to apply drought stress treatments to rice at the seedling stage. By analyzing morphological indicators, OJIP curves, and JIP-test parameters, this study aims to reveal the response mechanisms of the photosynthetic electron transport chain in different rice cultivars at the seedling stage under drought stress, identify the tolerance thresholds of cultivars through comprehensive analysis, and explore the potential application of these physiological indicators in irrigation management, thereby providing a basis for breeding drought-tolerant varieties.

## 2. Results

### 2.1. Effects of Drought Stress on Morphological Indicators of Rice Leaves at Seedling Stage

**Figure 1:**
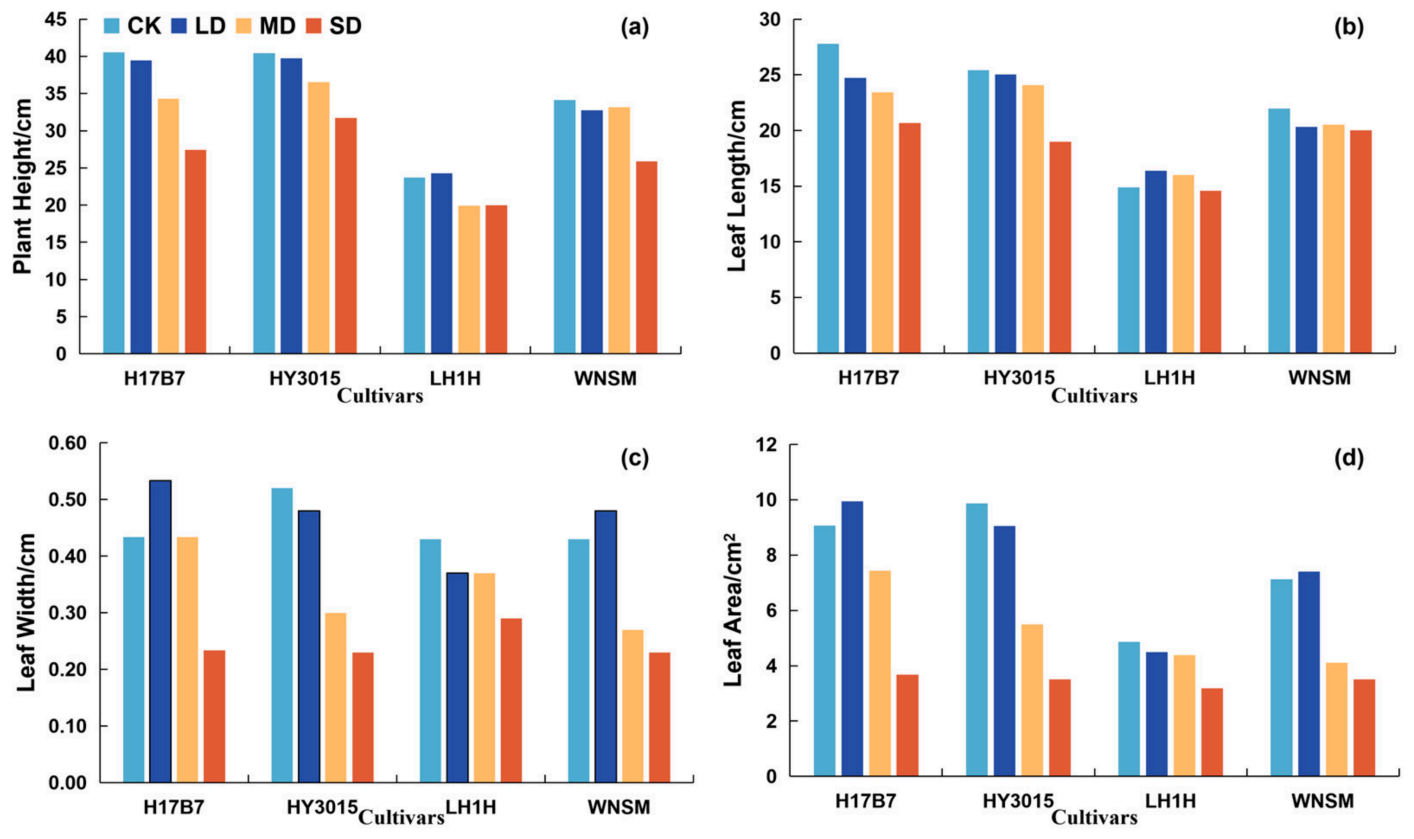
Changes in leaf morphological indicators of rice seedlings under drought stress. (**a**–**d**) represent changes in plant height, leaf length, leaf width, and leaf area of rice seedlings under drought stress, respectively. Data are presented as mean ± standard deviation (n = 3). Statistical comparisons among treatments were performed using two-way ANOVA (variety × drought treatment) followed by post hoc tests; detailed results of the main effects and simple effects are described in the Results section.

As shown in Figure 1, the plant height, leaf length, leaf width, and leaf area of the four rice cultivars exhibited a downward trend following drought stress. Two-way ANOVA revealed that the variety × drought interaction was not significant for the plant height and leaf length (*p* > 0.05), but was significant for leaf width and leaf area (*p* < 0.05). Therefore, the plant height and leaf length were analyzed for the main effect of drought treatment, while the leaf width and leaf area were subjected to simple effect tests within each variety.

For the plant height and leaf length, since the interaction was not significant, the effect of drought treatment was consistent across all varieties; therefore, data were pooled across varieties for main effect analysis. The main effect of drought treatment was significant: across all cultivars, the order of plant height and leaf length under different treatments was consistently CK > LD > MD > SD. Specifically, under SD treatment, the plant height and leaf length of H17B7 decreased by 32.40% and 25.57%, respectively, and the plant height of HY3015 decreased by 21.59% compared to CK, all reaching statistical significance (*p* < 0.05). Although slight increases in the plant height and leaf length were observed for LH1H and WNSM under LD or MD, these variations did not lead to a significant interaction effect.

For the leaf width and leaf area, as the interaction was significant, simple effect tests were conducted within each variety. The response patterns varied among the cultivars: H17B7 and WNSM showed a slight increase under LD followed by a subsequent decrease, whereas HY3015 and LH1H exhibited a continuous decline with intensifying drought. Among the four varieties, LH1H showed the smallest reduction, while compared to CK, the leaf width and leaf area under SD decreased by 32.56% and 34.70%, respectively.

### 2.2. Effects of Drought Stress on SPAD and Chlorophyll Content of Rice Leaves at Seedling Stage

As shown in Table 1, drought stress induced dynamic changes in leaf SPAD values and chlorophyll content of rice seedlings at the seedling stage, with responses varying by cultivar and stress intensity, although the overall trend was a decline.

For SPAD values, H17B7, HY3015, and LH1H showed no significant differences across all drought treatments compared with the control. WNSM exhibited slight decreases under LD and MD, but these were not significantly different from CK; however, a sharp and significant reduction occurred under SD.

For chlorophyll content, H17B7 showed a significant decrease even under mild drought, with further substantial declines under moderate and severe drought, and no significant difference between the latter two, indicating that its chlorophyll accumulation was impaired at mild stress. HY3015 and WNSM showed similar responses, with no significant difference between mild drought and CK, while both moderate and severe treatments led to significantly lower chlorophyll content than CK. LH1H displayed a precise stepwise decreasing pattern, with CK significantly higher than mild and moderate treatments, and severe stress significantly lower than all other treatments.

### 2.3. Effects of Drought Stress on Chlorophyll Fluorescence Characteristics of Rice Leaves at Seedling Stage

#### 2.3.1. Effects of Drought Stress on OJIP Curves of Rice Leaves at Seedling Stage

**Figure 2:**
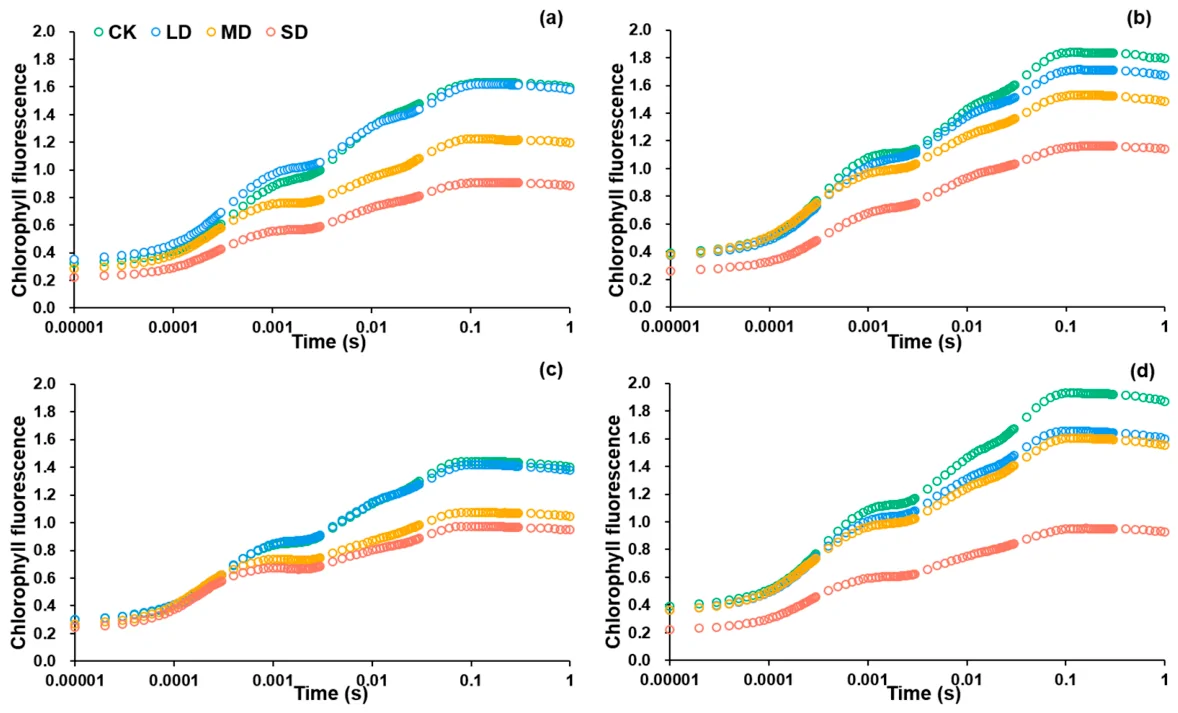
Changes in OJIP curves of rice leaves at the seedling stage under drought stress. (**a**–**d**) represent varieties H17B7, HY3015, LH1H, and WNSM, respectively. Each data point on the curves represent the mean of valid measurements after outlier removal.

As shown in Figure 2, the OJIP curves of rice seedling leaves for the four cultivars under drought stress exhibited a typical polyphasic rise, including the four basic steps: O, J, I, and P. However, as the intensity of drought increased, the OJIP curves tended to flatten, indicating that drought stress caused damage to the rice seedling leaves. The impacts of drought stress on H17B7 and LH1H were more pronounced under MD and SD treatments. Compared with CK, the chlorophyll fluorescence intensities at the O, J, I, and P phases of H17B7 decreased by 9.85%, 19.47%, 26.70%, and 25.24% under MD treatment, and by 30.32%, 39.90%, 45.03%, and 44.36% under SD treatment, respectively. For LH1H, these intensities decreased by 5.84%, 15.35%, 24.19%, and 25.50% under MD treatment, and by 13.70%, 22.81%, 31.57%, and 32.61% under SD treatment. Both HY3015 and WNSM showed decreases across all treatments, with minor reductions under LD and MD, and more substantial reductions under SD treatment, decreasing by 33.67%, 35.01%, 35.56%, and 36.75% and 42.29%, 46.13%, 49.63%, and 50.62%. The rise in the O-J phase is related to the closure of a portion of the PSII reaction centers. The inhibition of the O-J rise has been linked to a decline in the reduction level of the primary electron acceptor quinone (Q_A_^−^) [40,41], suggesting a decrease in the electron capture rate and a reduction in the speed at which Q_A_^−^ is oxidized by Q_B_ and other electron transport chain members under drought stress [42]. This phenomenon was more evident under the SD treatment for H17B7, HY3015, and WNSM. Based on the above analysis, drought primarily affected the J-I and I-P segments of the OJIP curves. The restricted rise in the O-J, J-I, and I-P segments suggests that drought stress inhibited the PSII acceptor side, the PQ pool, and the PSI acceptor side, thereby demonstrating the impact of drought stress on both PSII and PSI.

**Figure 3:**
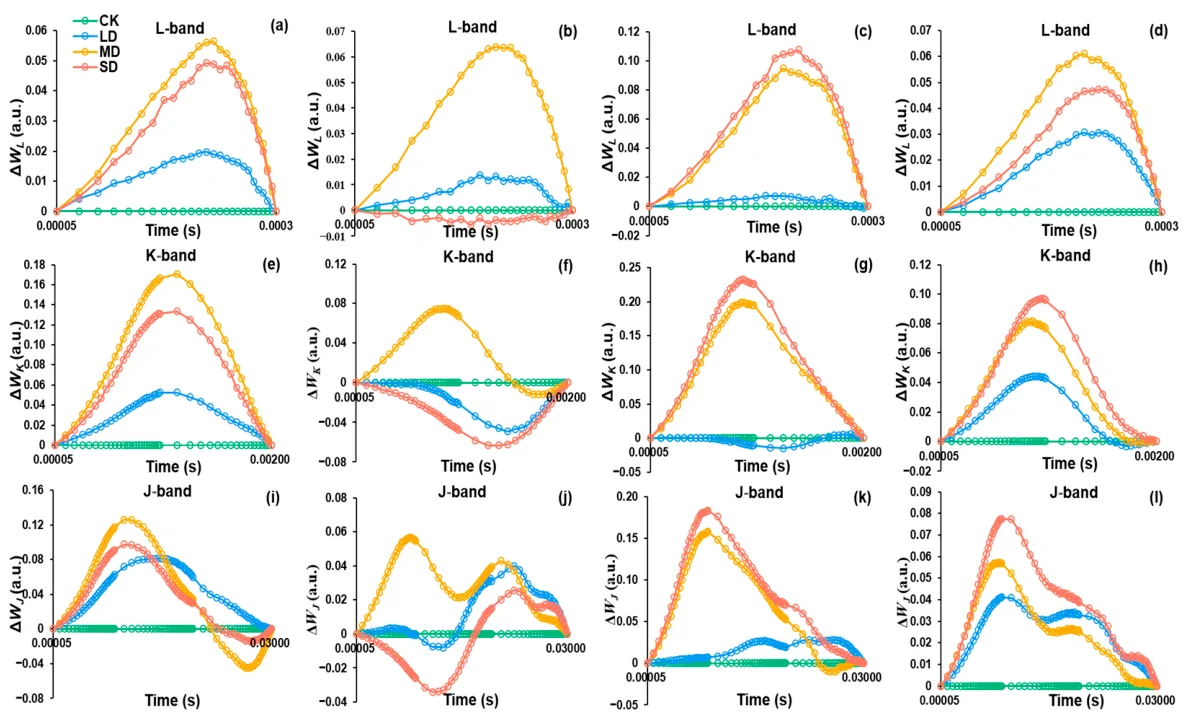
Changes in Δ*W_L_* (L-band), Δ*W_K_* (K-band), and Δ*W_J_* (J-band) of rice leaves at the seedling stage under drought stress. (**a**–**d**) represent the L-band of cultivars H17B7, HY3015, LH1H, and WNSM, respectively; (**e**–**h**) represent the K-band of cultivars H17B7, HY3015, LH1H, and WNSM, respectively; (**i**–**l**) represent the J-band of cultivars H17B7, HY3015, LH1H, and WNSM, respectively. Each data point on the curves represent the mean of valid measurements after outlier removal.

To further investigate the impact of drought stress on the PSII reaction centers of rice leaves at the seedling stage, fluorescence parameters of the O-P phase were normalized. The results in Figure 3 show that, with the exception of the SD treatment for HY3015, the Δ*W_L_* values for all other treatments were significantly higher than those of the CK, and a positive L-band was observed. This indicates that drought stress reduced the connectivity of excitation energy between the antenna pigments and the PSII reaction centers, resulting in PSII damage. Previous studies have demonstrated that the appearance of the K-step is a hallmark of damage to the Oxygen-Evolving Complex (OEC) on the electron donor side of PSII [43]; simultaneously, Δ*W_K_* represents the degree of OEC destruction. A negative K-band was observed in the LD and SD treatments of HY3015 and the LD treatment of LH1H, suggesting that rice initiates a series of defense mechanisms under mild drought stress to avoid photodamage. However, when moderate drought stress is reached, the regulatory capacity is strongly impaired and OEC is damaged, resulting in substantial impairment of PSII under heavy drought. For H17B7, positive K-band appeared under all drought stress levels; the increase in Δ*W_K_* was greater under MD than under SD. In WNSM, a positive K-band appeared across all drought treatments, with Δ*W_K_* values increasing progressively as the drought stress intensified. Under drought stress, all four rice cultivars exhibited a positive J-band, indicating that drought stress inhibited the donor side of PSII; OEC damage resulted in an insufficient electron supply, thereby affecting the reoxidation of Q_A_^−^.

#### 2.3.2. Effects of Drought Stress on JIP-Test Parameters of Rice Leaves at Seedling Stage

**Figure 4:**
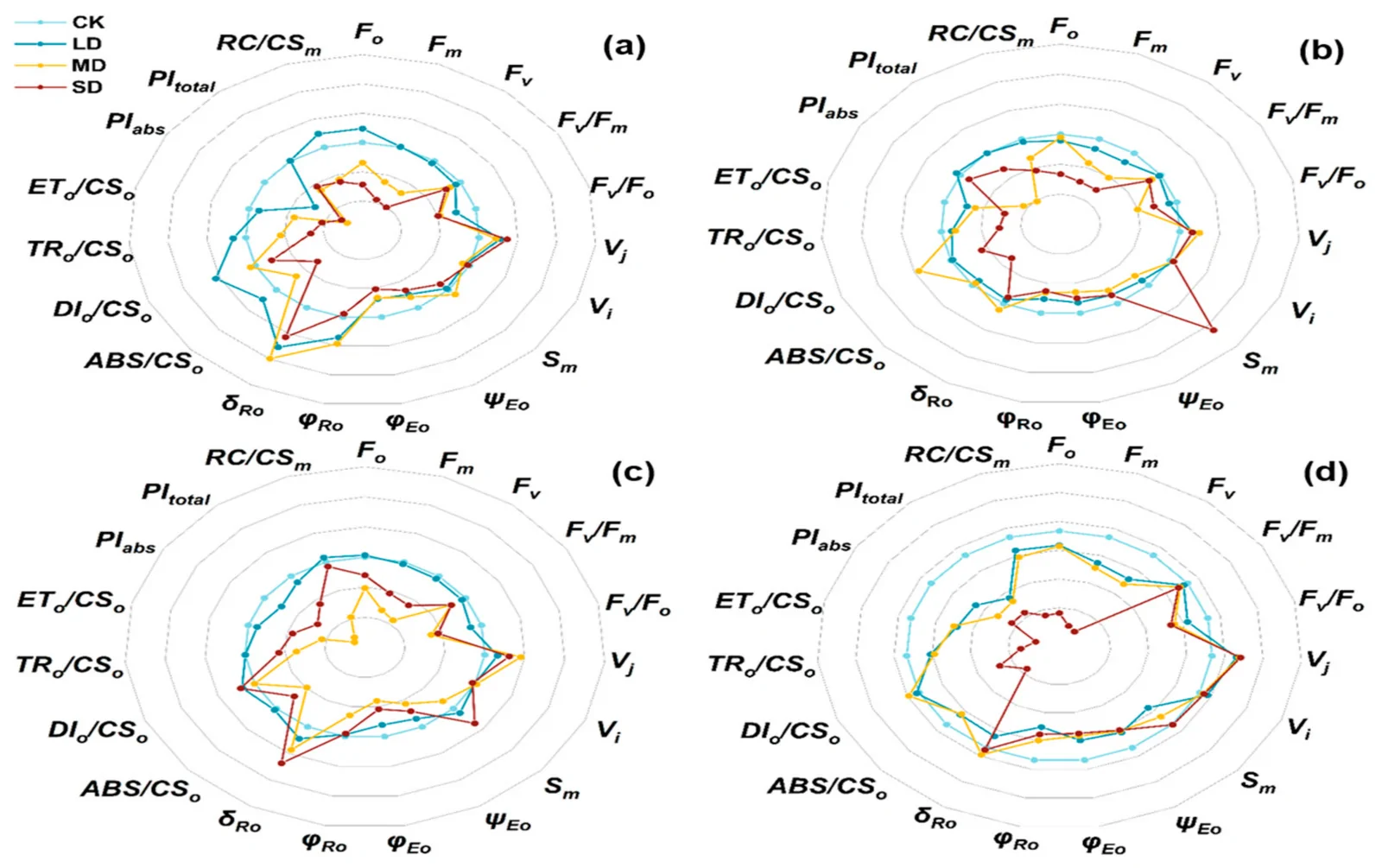
Changes in the JIP-test parameters of rice seedling leaves under drought stress. (**a**–**d**) represent the varieties H17B7, HY3015, LH1H, and WNSM, respectively. Each data point in the radar charts represents the mean of valid measurements after outlier removal.

The *F_v_*/*F_m_*, *PI_abs_*, and *PI_total_* of all four cultivars were lower than those of CK under the three drought stress treatments (Figure 4). The value of *F_v_*/*F_m_* is commonly used to measure the degree of external stress on plants [44]; its decline indicates that drought stress has impacted the photosynthetic system of rice leaves, leading to a reduction in overall photosynthetic output. In H17B7 under LD treatment, *TR_o_*/*CS_o_* and *DI_o_*/*CS_o_* increased while *ET_o_*/*CS_o_* decreased, suggesting that the energy trapped per cross-section increased, but the energy transferred decreased, with more energy being diverted to thermal dissipation. This indicates that drought stress weakened the electron transport efficiency. In HY3015 and WNSM, *TR_o_*/*CS_o_* and *ET_o_*/*CS_o_* exhibited a steady decline as drought stress intensified, indicating a progressive decline in PSII photochemical activity, reflecting a gradual loss of PSII function, which is consistent with the observed reductions in leaf SPAD values and growth. For LH1H, no significant differences were observed in *TR_o_*/*CS_o_*, *ET_o_*/*CS_o_*, and *DI_o_*/*CS_o_* under the LD treatment compared to CK, but these values dropped sharply under MD and SD. This indicates that the PSII reaction centers of LH1H were virtually unaffected under mild drought, but the photosynthetic system collapsed under moderate drought, which is consistent with this cultivar’s performance in plant height, OJIP curves, and L-bands. In HY3015, Sm increased sharply under SD treatment, indicating a slowed rate of electron transfer from Q_A_^−^ to downstream components and impaired function of the PSI acceptor side. The summary statistics of JIP-test parameters are shown in Table S1.

#### 2.3.3. Effects of Drought Stress on the State of PSII Reaction Centers

**Figure 5:**
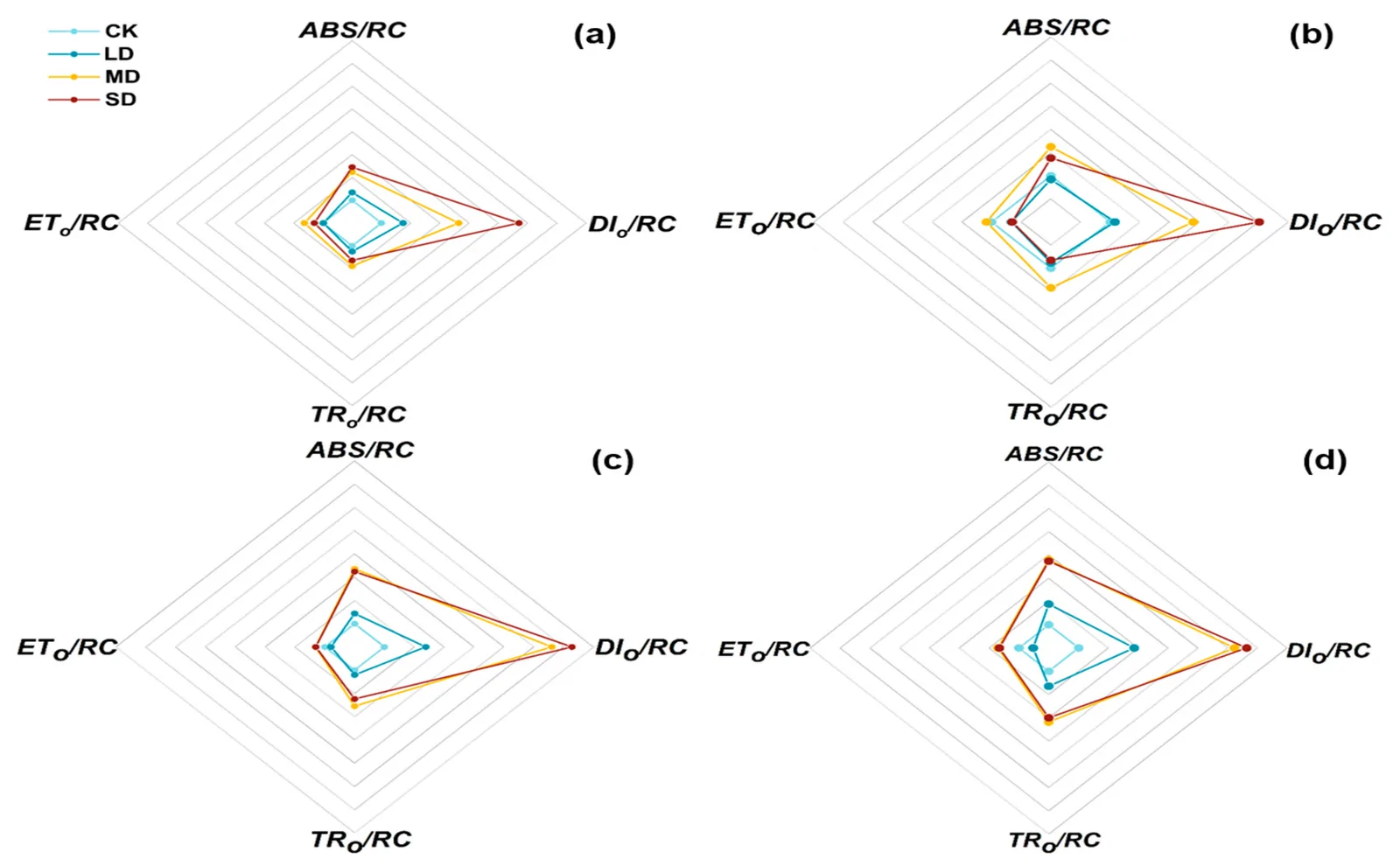
Changes in the PSII reaction center status in rice leaves at the seedling stage under drought stress. (**a**–**d**) represent the varieties H17B7, HY3015, LH1H, and WNSM, respectively. Each data point in the radar charts represent the mean of valid measurements after outlier removal.

The parameters *ABS*/*RC*, *TR_o_*/*RC*, *ET_o_*/*RC,* and *DI_o_*/*RC* constitute the energy distribution within the PSII unit reaction centers. As shown in Figure 5, analysis of these energy flow parameters per reaction center revealed that *ABS*/*RC*, *TR_o_*/*RC*, *ET_o_*/*RC*, and *DI_o_*/*RC* in all four cultivars exhibited an upward trend as the intensity of drought stress increased, with the increase in *DI_o_*/*RC* being the most significant. Concurrently, combined with the results in Figure 4, a decrease in *RC*/*CS_m_* was observed. This indicates that while the energy absorbed, trapped, transported, and dissipated by each active reaction center of PSII increased, the total number of active reaction centers decreased, which is consistent with the OJIP analysis results. Across all four cultivars, both *Ψ_Eo_* and *φ_Eo_* decreased following the occurrence of drought stress, indicating the inhibition of PQ pool reoxidation and overall damage to the photosystems. For H17B7 and LH1H, *δ_Ro_* increased substantially, while it remained largely unchanged for HY3015 and WNSM. This suggests that the PSI and the downstream electron transport chain in H17B7 and LH1H were significantly affected by the stress.

### 2.4. Principal Component Analysis of JIP-Test Parameters in Rice Leaves at Seedling Stage Under Drought Stress

**Figure 6:**
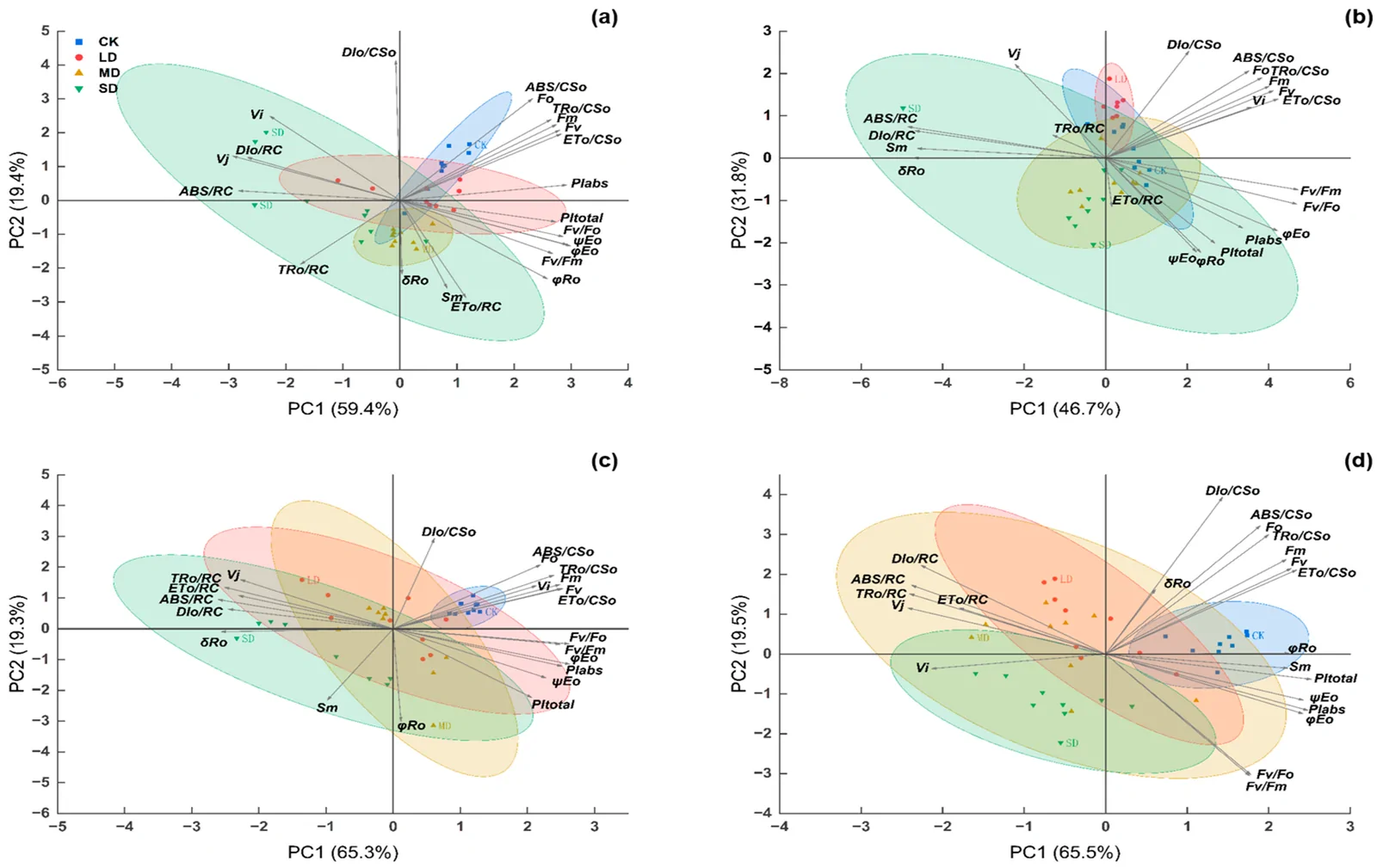
PCA of the JIP-test parameters in rice leaves at the seedling stage under drought stress. (**a**–**d**) represent the varieties H17B7, HY3015, LH1H, and WNSM, respectively.

To analyze the photosystem stress induced by changes in photosynthetic reactions under drought conditions, 22 JIP-test parameters, including *F_v_*/*F_m_*, *PI_abs_* and *PI_total_*, were selected. Principal Component Analysis (PCA) was performed on the JIP-test parameters of the four rice cultivars following drought stress, as shown in Figure 6. Based on the criteria of eigenvalues > 1 and a cumulative contribution rate > 85%, three principal components were extracted for H17B7, HY3015, LH1H, and WNSM, with cumulative contribution rates of 88.27%, 92.36%, 92.88%, and 94.35%, respectively. The first two components accounted for 78.8%, 78.5%, 84.6%, and 80.0% of the total variance, effectively explaining the primary differences in parameters among treatments. Specifically, PC1 for H17B7 explained 59.4% of the variation. Across the four cultivars, the CK group was clearly distinguished from drought treatments; however, no distinct clustering occurred among drought intensities, indicating that the measured fluorescence parameters did not clearly resolve stress severity. Since PC1 captures the most significant information from the original data [45], the parameters influencing its formation were further analyzed. In all four cultivars, PC1 was negatively correlated with *V_i_*, *ABS*/*RC*, *DI_o_*/*RC,* and *TR_o_*/*RC*, and positively correlated with *F_o_*, *F_m_*, *F_v_*, *F_v_*/*F_m_*, *F_v_*/*F_o_*, *ψ_Eo_*, *φ_Eo_*, *ABS*/*CS_o_*, *TR_o_*/*CS_o_*, *ET_o_*/*CS_o_*, *PI_abs_*, and *PI_total_*. The parameters *V_i_*, *S_m_*, *ET_o_*/*RC*, *φ_Ro_*, *δ_Ro_*, and *DI_o_*/*CS_o_* exhibited varying patterns across the four cultivars, suggesting that each cultivar adopts different coping strategies when subjected to drought stress.

### 2.5. Correlation Analysis Between Physicochemical and JIP-Test Parameters of Rice Leaves Under Drought Stress

**Figure 7:**
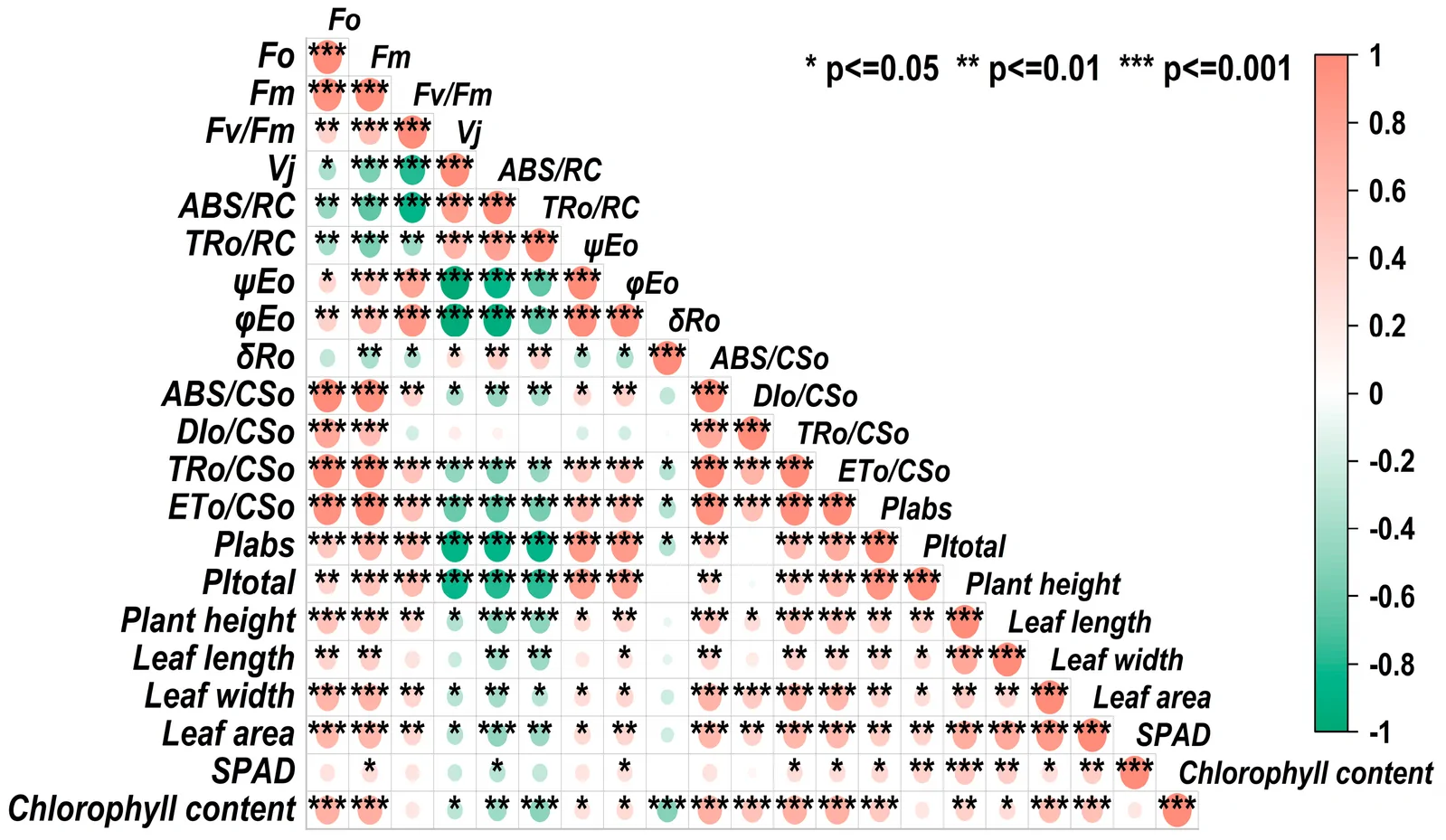
Correlation plot of the leaf physicochemical parameters and JIP-test parameters in rice seedlings under drought stress. The color of each square represents the Pearson correlation coefficient, with orange indicating a positive correlation and green indicating a negative correlation; darker colors denote stronger correlations. Asterisks indicate the level of statistical significance: * *p* ≤ 0.05 (significant), ** *p* ≤ 0.01 (highly significant), and *** *p* ≤ 0.001 (extremely significant). The correlation matrix was generated using data pooled from all four varieties (H17B7, HY3015, LH1H, and WNSM) rather than separately for each cultivar; therefore, no subpanels (a–d) are shown.

As shown in Figure 7, plant height, leaf width, leaf area, and chlorophyll content exhibited extremely significant positive correlations with *F_o_*, *F_m_*, *ABS*/*CS_o_*, *TR_o_*/*CS_o_*, and *ET_o_*/*CS_o_*. Chlorophyll content also showed extremely significant positive correlation with *DI_o_*/*CS_o_*. Conversely, plant height was extremely significantly negatively correlated with *ABS*/*RC*, and chlorophyll content was highly significantly negatively correlated with *δ_Ro_*. No extremely significant correlations were observed between the leaf length or SPAD and any of the chlorophyll fluorescence parameters. The results of the correlation analysis revealed that morphological growth traits covaried with selected fluorescence parameters under drought stress. As the seedling stage is a period of vegetative growth, the simultaneous increase in *F_o_*, *F_m_*, and *ABS*/*RC* under drought stress implies the inactivation of PSII reaction centers. Furthermore, the increase in *DI_o_*/*CS_o_* and the decrease in *δ_Ro_* occurring alongside changes in chlorophyll content suggest that electron transport downstream of PSI is also obstructed. Consequently, the photosystems are no longer able to effectively utilize the captured energy, which must instead be dissipated as heat.

## 3. Discussion

Studies have shown that drought stress significantly impacts the morphological indicators of rice plants [46,47]. In this study, the plant height, leaf length, leaf width, leaf area, SPAD, and chlorophyll content of rice at the seedling stage generally followed a downward trend under drought stress. Two-way ANOVA revealed that the variety × drought interaction was not significant for the plant height and leaf length, with consistent declines across the varieties and the largest reductions under SD; however, significant interactions were observed for the leaf width and leaf area, with distinct response patterns among cultivars. For H17B7, the plant height and leaf length began to decrease from LD and showed significant differences under SD; however, its SPAD values remained stable across all treatments, whereas chlorophyll content decreased significantly even under LD, indicating that morphological growth is sensitive to drought while chlorophyll responds earlier than SPAD within the photosynthetic pigment system. HY3015, LH1H, and WNSM exhibited non-significant slight increases in some morphological parameters under LD, suggesting a compensatory growth effect in rice under mild drought stress, consistent with the findings of Yi [48] and Li et al. [49]. LH1H showed the smallest reductions in the leaf width and leaf area under SD (32.56% and 34.70%, respectively); together with its stable SPAD values, this further supports that LH1H possesses relatively strong drought tolerance. In contrast, SPAD of WNSM only decreased sharply under SD, while chlorophyll was already impaired at MD, indicating that chlorophyll is more sensitive to drought than SPAD in this cultivar, with different physiological parameters exhibiting distinct response thresholds to stress. It should be noted that the response patterns of SPAD readings and chemically extracted chlorophyll content were not entirely consistent. This discrepancy arises from fundamental methodological differences, as SPAD is influenced by leaf thickness, water content, and other factors, all of which can be altered under drought conditions [50]; chemical extraction, by contrast, provides an absolute quantitative value that is independent of leaf physical status.

Chlorophyll fluorescence is a widely used non-invasive probe for studying plant photosynthesis, which can effectively reflect the primary photochemical reactions of photosystem II and the activity of photosynthetic electron transport [51]. In this study, drought stress led to the inhibition of the polyphasic rise in the OJIP curves. For LH1H, the curve under LD treatment largely overlapped with the CK, but the J-I and I-P segments decreased significantly under MD and SD. This indicates an upper limit to drought tolerance; once the stress exceeds the tolerance threshold, the PQ pool and the PSI acceptor side sustain substantial impairment, consistent with the findings of Schansker et al. [52]. LH1H exhibited the least inhibition of the OJIP rise across all stress treatments, followed by HY3015, demonstrating superior stability. In WNSM, the P-peak decreased by more than 50% under SD, indicating a severe obstruction of electron transport on the PSI acceptor side. The appearance of distinct positive L-bands, K-bands, and J-bands signifies reduced energy connectivity between PSII units, inactivation of the OEC, and accumulation of the primary electron acceptor Q_A_^−^ [42,53,54]. For LH1H, the L-band, K-band, and J-band were inconspicuous under LD, while *TR_o_*/*CS_o_*, *ET_o_*/*CS_o_*, and *DI_o_*/*CS_o_* remained stable, further validating its higher drought tolerance threshold and stable photosystem structure under mild stress. Beyond light drought, however, Δ*W_L_*, Δ*W_K_*, and Δ*W_J_* increased sharply, indicating a severe impairment of the photosystem. Under identical stress conditions, the increases in Δ*W_K_* and Δ*W_J_* for H17B7 were higher than that of Δ*W_L_*, suggesting that drought stress exerts more severe inhibition on the PSII donor side and the PQ pool. For H17B7, the increase in Δ*W_K_* was greater under MD than under SD. This pattern suggests that the rice plant may initiate adaptive regulatory mechanisms when OEC is partially damaged under moderate drought; however, once the stress intensity exceeds the regulatory capacity, OEC damage reaches a peak, eventually leading to the inactivation of a large number of PSII reaction centers. Such a response has been observed in other studies on drought-stressed rice, indicating a threshold-dependent behavior of PSII photoprotection under progressive water deficit.

The correlation analysis in this study revealed a covariation between morphological growth traits and photosystem function; under drought, the increase in *ABS*/*CS_o_* likely reflected a reduction in the number of active reaction centers and the associated energy redistribution. Although *ABS*/*CS_o_* increased, the elevation of *F_o_* and *F_m_* under drought stress represents the partial inactivation of PSII reaction centers, preventing the effective conversion of *ABS*/*CS_o_*. Furthermore, the negative correlation between the plant height and *ABS*/*RC* suggests that the remaining active reaction centers were inhibited and their efficiency reduced. To avoid more severe damage, the system must dissipate the energy that cannot be utilized effectively as heat (*DI_o_*/*CS_o_*).

This study has certain limitations. Key gas exchange parameters (e.g., Pn, Gs, Ci) and water relation traits (e.g., WUE, RWC) were not measured, limiting a full assessment of the photosynthetic response to drought stress. Future research incorporating these indices, combined with transcriptomic analysis to examine expression differences in genes related to PQ pool repair, would offer a more comprehensive understanding of the underlying mechanisms and provide new insights.

## 4. Materials and Methods

### 4.1. Experimental Materials

Four rice cultivars—H17B7, HY3015, LH1H, and WNSM—were selected and pro-vided by the Fujian Engineering and Technology Research Center for Special Crop Breeding and Utilization. The experiment was conducted in the Smart Crop Factory of the College of Agriculture, Fujian Agriculture and Forestry University, where the temperature was maintained at 28 °C. Before transplanting, seeds were germinated and seedlings were raised in a controlled-environment growth chamber set at a constant temperature of 28 °C, relative humidity of 70%, and a photoperiod of 12 h light/12 h dark. After transplanting, the seedlings were transferred to the Smart Crop Factory, where they were grown under natural sunlight without supplemental lighting. The plastic pots measured 12 cm in height and 10 cm in diameter. The experimental soil was paddy soil with the following physicochemical properties: pH 5.54, available phosphorus 35.00 mg/kg, available potassium 105.00 mg/kg, alkaline hydrolyzable nitrogen 121.30 mg/kg, and a water content of 31.00%. At the time of transplanting, the soil was prepared as a 3:1 mixture of paddy soil and nutrient soil. No additional fertilizer was applied during the experimental period.

### 4.2. Experimental Design

An artificial pot-based water control method was employed. The experiment followed a split-plot design, with water treatments as main plots and cultivars as subplots. Within each main plot, pots of the same cultivar were placed consecutively in groups of three (representing three replicate pots), and the four cultivar groups were arranged side by side. Each treatment included three replicate main plots (12 main plots in total); these main plots were randomly arranged on the greenhouse bench, and their positions were periodically rotated. For each cultivar, 120 seeds were selected for soaking and germination. After germination, seeds were sown into seedling trays. At the three-leaf-one-heart stage, 36 uniform seedlings per cultivar were transplanted into non-perforated pots, three plants per pot. After 2–3 days of recovery, drought treatments were initiated: CK (2–3 cm water layer), LD (80 ± 5% field capacity), MD (60 ± 5% field capacity), and SD (40 ± 5% field capacity). Drought lasted for 7 days. Soil moisture was monitored and adjusted twice daily (08:00 and 17:00) by weighing each pot and replenishing water to the target weight. The three plants within each pot were treated as subsamples; data were averaged at the pot level prior to statistical analysis.

### 4.3. Experimental Methods

#### 4.3.1. Agronomic Traits

Measurements were conducted using physical methods. Each pot served as one biological replicate. The plant height, leaf length, leaf width, and leaf area were determined, and the measurements from the three plants within the same pot were averaged to represent the value of that pot. Each treatment included three replicates (pots). Plant height was measured as the length from the soil surface to the leaf tip. Leaf length was measured from the leaf collar to the leaf tip, and leaf width was measured at the widest part of the fully expanded leaf. Leaf area was calculated by the length-width correction method using Formula (1), with the correction coefficient K set to 0.75 [55,56].

Leaf area (cm^2^) = length × width × K (1)

#### 4.3.2. Chlorophyll Content and SPAD Index

Relative chlorophyll content (SPAD value) was determined using a SPAD-502 chlorophyll meter (Konica Minolta, Osaka, Japan) in a non-destructive manner. Three functional leaves with uniform growth and no pests or diseases were selected per plant. Three experimental points were chosen on each leaf at intervals of 1–2 cm, totaling 27 measurement points per pot. The absolute chlorophyll content was determined using the ethanol extraction method [57,58]. In this paper, the term “chlorophyll content” specifically refers to the absolute chlorophyll content obtained by extraction. Fresh leaf samples (0.2 g) were taken from each pot and placed into 10 mL centrifuge tubes, to which 10 mL of 95% ethanol was added. The tubes were kept at 4 °C in the dark for 48 h, during which they were shaken periodically until the leaf samples became completely white. The extract was then used for spectrophotometric analysis. Absorbance values were recorded at 665 nm, 649 nm, and 470 nm, with 95% ethanol serving as the blank. The content of chlorophyll a (Ca), chlorophyll b (Cb), and total chlorophyll (Ct) were calculated using Formulas (2)–(4):

Ca (mg·g^−1^ FW) = 13.95 × A665 − 6.88 × A649 (2)

Cb (mg·g^−1^ FW) = 24.96 × A649 − 7.32 × A665 (3)

Ct (mg·g^−1^ FW) = Ca + Cb (4)

#### 4.3.3. OJIP Curves and Parameters

Chlorophyll fluorescence parameters were measured using a Handy PEA portable plant efficiency analyzer (Hansatech Instruments, King’s Lynn, UK). The saturating pulse intensity was set to 3500 μmol·m^−2^·s^−1^, with a pulse duration of 1 s. Prior to measurement, rice plants were dark-adapted for 30 min. After dark adaptation, three fully expanded functional leaves were selected per plant, avoiding the main vein, and three measurement points were selected on each leaf at intervals of 1–2 cm. A total of 81 raw readings were obtained per cultivar per treatment (3 plants × 3 leaves × 3 spots × 3 replicates). Readings with instrument self-check errors or outliers were excluded, and the mean for each treatment were calculated based on the valid data. The measured parameters included *F_o_*, *F_m_*, *F_v_*, *F_v_*/*F_m_*, *F_v_*/*F_o_*, *V_j_*, *V_i_*, *S_m_*, *ψ_Eo_*, *φ_Eo_*, *φ_Ro_*, *δ_Ro_*, *ABS*/*CS_o_*, *TR_o_*/*CS_o_*, *ET_o_*/*CS_o_*, *DI_o_*/*CS_o_*, *ABS*/*RC*, *TR_o_*/*RC*, *ET_o_*/*RC*, *DI_o_*/*RC*, *RC*/*CS_m_*, *PI_abs_*, and *PI_total_*. Among these, *RC*/*CS_m_* was calculated using Formulas (5) and (6) [36]. The parameters and their definitions are listed in Table 2. Characteristic time points were designated as follows: O at 50 µs, K at 300 µs, J at 2 ms, I at 30 ms, and P at 300 ms. OJIP curves were plotted based on the output data. O-K, O-J, and O-I phases of the OJIP curves were normalized, and the three main bands occurring from the O-phase to the P-phase (L-band, K-band, and J-band) were calculated using Formulas (7)–(12) [37]. Measurements were conducted between 8:00 and 10:00 AM.

*RC*/*CS_m_* = *F_m_* × *φ_Po_* × *V_j_*/*M_o_* (5)

*M_o_* = 4 × (*F_k_* − *F_o_*)/(*F_m_* − *F_o_*) (6)

V_OK_ = (*F_t_* − *F_o_*)/(*F_K_* − *F_o_*) (7)

V_OJ_ = (*F_t_* − *F_o_*)/(*F_J_* − *F_o_*) (8)

V_OI_ = (*F_t_* − *F_o_*)/(*F_I_* − *F_o_*) (9)

L-band: Δ*W_L_* = V_OK(LD/MD/SD)_ − V_OK(CK)_ (10)

K-band: Δ*W_K_* = V_OJ(LD/MD/SD)_ − V_OJ(CK)_ (11)

J-band: Δ*W_J_* = V_OI(LD/MD/SD)_ − V_OI(CK)_ (12)

### 4.4. Statistical Analysis

A two-way analysis of variance (two-way ANOVA) was performed to test the effects of variety, drought treatment, and their interaction on each parameter. When the interaction was significant (*p* < 0.05), Bonferroni-corrected simple effect analyses were conducted within each variety; when the interaction was not significant (*p* > 0.05), Tukey’s HSD post hoc test was used to compare main effect means. Correlation analysis was performed using Pearson correlation coefficients. PCA was conducted with an auto-scaling mode for data standardization. Statistical analyses were carried out using SPSS 27, and figures were generated using Origin 2025 and Microsoft Excel 2016.

## 5. Conclusions

In conclusion, drought stress affects both the morphogenesis and photosynthesis of rice. Beyond the drought tolerance threshold, drought stress mainly impairs the function of the photosynthetic electron transport chain at the PQ pool and PSI acceptor side in rice seedling leaves, thereby affecting morphological indicators and chlorophyll content, with the degree varying by cultivar. LH1H demonstrated strong drought tolerance. HY3015 and WNSM showed lower tolerance thresholds, with their photosystems affected even under mild drought; however, their systems remained well-coordinated, with functions declining steadily. H17B7 was the most sensitive to drought stress, exhibiting severe photosystem damage and a disorganized response pattern even under mild drought conditions.

## Figures and Tables

**Table 1:** Changes in leaf SPAD values and chlorophyll content in rice seedlings under drought stress.

Varieties	Treatment	SPAD Value	Chlorophyll Content (mg·g^−1^)
H17B7	CK	21.81 ± 2.65 a ^1^	1.44 ± 0.01 a
	LD	19.76 ± 1.50 a	0.93 ± 0.11 b
	MD	20.32 ± 1.72 a	0.60 ± 0.07 c
	SD	18.26 ± 1.67 a	0.66 ± 0.06 c
HY3015	CK	18.07 ± 1.28 a	0.92 ± 0.02 a
	LD	19.76 ± 2.17 a	0.94 ± 0.01 a
	MD	19.40 ± 2.23 a	0.62 ± 0.06 b
	SD	17.41 ± 1.47 a	0.57 ± 0.01 b
LH1 H	CK	15.19 ± 1.25 a	1.31 ± 0.02 a
	LD	14.77 ± 1.67 a	0.67 ± 0.02 b
	MD	14.21 ± 2.22 a	0.56 ± 0.03 b
	SD	15.40 ± 0.82 a	0.45 ± 0.08 c
WNSM	CK	21.60 ± 1.19 a	0.95 ± 0.04 a
	LD	21.20 ± 2.23 a	0.89 ± 0.04 a
	MD	19.27 ± 1.55 a	0.40 ± 0.02 b
	SD	14.97 ± 0.66 b	0.43 ± 0.13 b

^1^ Data are presented as mean ± standard deviation (n = 3). Different letters indicate significant differences among drought treatments within the same cultivar (*p* < 0.05, Bonferroni correction); the same letter indicates no significant difference (*p* > 0.05). Due to significant variety × drought treatment interactions (two-way ANOVA), these letters denote differences only within each cultivar.

**Table 2:** Chlorophyll fluorescence parameters and interpretations.

Parameters	Parameter Interpretation
*F_o_*	Initial fluorescence intensity
*F_m_*	Maximum fluorescence intensity
*F_v_*	Variable fluorescence intensity
*F_v_*/*F_m_*	Maximum quantum efficiency of PSII
*F_v_*/*F_o_*	Potential activity of PSII
*V_j_*	Relative variable fluorescence intensity at the J-step
*V_i_*	Relative variable fluorescence intensity at the I-step
*S_m_*	Electron acceptor pool capacity on the receptor side of PSII
*M_o_*	Initial slope of relative fluorescence
*ψ_Eo_*	Probability that a trapped exciton moves an electron into the trapped electron transport chain beyond Q_A_^−^
*φ_Eo_*	Quantum yield of electron transport
*φ_Ro_*	Quantum yield for the reduction in end acceptors of PSI per photon absorbed
*δ_Ro_*	Efficiency with which an electron can move from the reduced intersystem electron acceptors to the PSI end electron acceptors
*ABS*/*CS_o_*	Absorbed energy flux per cross-section
*TR_o_*/*CS_o_*	Trapped energy flux per cross-section
*ET_o_*/*CS_o_*	Electron transport flux per cross-section
*DI_o_*/*CS_o_*	Dissipated energy flux per cross-section
*ABS*/*RC*	Absorption flux per reaction center
*TR_o_*/*RC*	Trapped energy flux per reaction center
*ET_o_*/*RC*	Electron transport flux per reaction center
*DI_o_*/*RC*	Dissipated energy flux per reaction center
*RC*/*CS_m_*	Density of reaction centers per cross-section
*PI_abs_*	Performance index on absorption basis
*PI_total_*	Performance index for electron transport to PSI end acceptors

## Data Availability

Data for this paper can be obtained by contacting the corresponding author of this paper.

## Supplementary Materials

The following supporting information can be downloaded at: https://www.mdpi.com/article/10.3390/plants15162499/s1, Table S1. Summary statistics of JIP-test parameters in rice seedling leaves under drought stress.
