# Supplementary material for: Impact of Drought Stress on Photosynthetic Electron Transport in Rice Seedlings
Source: Plants (Basel). 2026 Aug 18;15(16):2499. doi: 10.3390/plants15162499 (PMC13516311; doi:10.3390/plants15162499)
Supplement: Supplementary file 1 [file plants-15-02499-s001.zip › plants-4404368-supplementary.pdf]

Table S1. Summary statistics of JIP-test parameters in rice seedling leaves under drought stress.

		F_o	F_m	F_v	F_v/F_m	F_v/F_o	V_j
H17B7	CK	1	1	1	1	1	1
	LD	1.095057	1.003244	0.981237	0.970565	0.895859	1.110414
	MD	0.861386	0.750109	0.723436	0.935744	0.810072	1.08741
	SD	0.713172	0.621395	0.599396	0.912118	0.800788	1.14517
HY3015	CK	1	1	1	1	1	1
	LD	0.956597	0.930584	0.923895	0.989618	0.960872	1.06347
	MD	0.979742	0.83114	0.792934	0.945699	0.798754	1.097878
	SD	0.733673	0.699406	0.690596	0.929746	0.882586	1.062437
LH1H	CK	1	1	1	1	1	1
	LD	1.012954	0.984797	0.977854	0.978259	0.944006	1.063773
	MD	0.794078	0.661267	0.628516	0.915747	0.741812	1.180146
	SD	0.879575	0.778899	0.754073	0.914299	0.777102	1.122288
WNSM	CK	1	1	1	1	1	1
	LD	0.926114	0.858773	0.842355	0.980845	0.917955	1.096141
	MD	0.921092	0.831621	0.809808	0.964044	0.865538	1.106559
	SD	0.572145	0.511565	0.496796	0.957058	0.850697	1.112095

		V_i	S_m	ψ_{Eo}	φ_{Eo}	φ_{Ro}	δ_{Ro}
H17B7	CK	1	1	1	1	1	1
	LD	0.975857	0.986797	0.897426	0.874752	1.141889	1.307713
	MD	0.961914	1.046429	0.918796	0.867065	1.184441	1.395504
	SD	0.990144	0.942733	0.865137	0.807179	0.977922	1.22907
HY3015	CK	1	1	1	1	1	1
	LD	1.017458	0.954849	0.93632	0.930127	0.90489	0.971794
	MD	1.016996	0.907007	0.901798	0.858317	0.862781	1.048108
	SD	1.01877	1.442194	0.937357	0.899946	0.851516	0.953105
LH1H	CK	1	1	1	1	1	1
	LD	0.998467	1.043831	0.938301	0.922172	0.985692	1.089924
	MD	1.007803	0.927776	0.825713	0.760223	0.857704	1.172966
	SD	0.986344	1.144888	0.881692	0.816473	0.979503	1.274742
WNSM	CK	1	1	1	1	1	1
	LD	1.032378	0.87263	0.91068	0.895685	0.825757	0.932952
	MD	1.01423	0.940529	0.901002	0.872902	0.896606	1.040728
	SD	1.01881	1.002955	0.895858	0.859541	0.864925	1.012805

		ABS/CS$_o$	*DI*$_o$*/CS*$_o$	*TR*$_o$*/CS*$_o$	*ET*$_o$*/CS*$_o$	*PIabs*	*PItotal*	*RC/CS*$_m$
H17B7	CK	1	1	1	1	1	1	1
	LD	1.095057	1.221102	1.064705	0.947562	0.690656	1.003082	1.096625
	MD	0.861386	1.026537	0.821618	0.760932	0.493484	0.746388	0.766843
	SD	0.713172	0.908044	0.666247	0.613514	0.527895	0.778463	0.750394
HY3015	CK	1	1	1	1	1	1	1
	LD	0.956597	0.991458	0.94755	0.884077	1.022854	0.99955	0.979143
	MD	0.979742	1.175306	0.928989	0.841548	0.622349	0.592517	0.865334
	SD	0.733673	0.831857	0.708192	0.690048	0.947659	0.864816	0.777654
LH1H	CK	1	1	1	1	1	1	1
	LD	1.012954	1.069473	0.99898	0.954962	0.897328	0.948299	1.030894
	MD	0.794078	1.001257	0.742854	0.62143	0.462462	0.484834	0.612935
	SD	0.879575	1.076638	0.830852	0.772899	0.681609	0.764331	0.967797
WNSM	CK	1	1	1	1	1	1	1
	LD	0.926114	1.009673	0.905655	0.813779	0.790597	0.71825	0.92778
	MD	0.921092	1.043522	0.891116	0.825669	0.686759	0.697014	0.889534
	SD	0.572145	0.65413	0.552071	0.495413	0.62231	0.62153	0.569932

		ABS/RC	*DI*$_o$*/RC*	*TR*$_o$*/RC*	*ET*$_o$*/RC*
H17B7	CK	1	1	1	1
	LD	1.171732	1.367577	1.122904	0.991715
	MD	1.618528	2.320481	1.443531	1.316639
	SD	1.724546	3.353038	1.318554	1.14716
HY3015	CK	1	1	1	1
	LD	0.968093	1.031965	0.951483	0.86569
	MD	1.24981	1.56012	1.169087	1.03439
	SD	1.152536	2.005005	0.93077	0.860871
LH1H	CK	1	1	1	1
	LD	1.087071	1.276437	1.04002	0.957686
	MD	1.470881	2.121378	1.309259	1.06219
	SD	1.448249	2.256271	1.247492	1.059997
WNSM	CK	1	1	1	1
	LD	1.088735	1.186595	1.064267	0.951986
	MD	1.279344	1.523085	1.218398	1.070434
	SD	1.273037	1.564273	1.200217	1.06565
